# PIEZO2 as the anomalous mechanotransducer channel in auditory hair cells

**DOI:** 10.1113/JP274996

**Published:** 2017-10-26

**Authors:** Maryline Beurg, Robert Fettiplace

**Affiliations:** ^1^ Department of Neuroscience University of Wisconsin School of Medicine and Public Health Madison WI 53706 USA

**Keywords:** cochlea, calcium, hair cells, single channels, development, mouse

## Abstract

Throughout postnatal maturation of the mouse inner ear, cochlear hair cells display at least two types of mechanically gated ion channel: normal mechanotransducer (MT) channels at the tips of the stereocilia, activated by tension in interciliary tip links, and anomalous mechanosensitive (MS) channels on the top surface of the cells. The anomalous MS channels are responsible for the reverse‐polarity current that appears in mutants in which normal transduction is lost. They are also seen in wild‐type hair cells around birth, appearing 2 days earlier than normal MT channels, and being down‐regulated with the emergence of the normal channels. We review the evidence that the normal and anomalous channels are distinct channel types, which includes differences in localization, susceptibility to pharmacological agents, single‐channel conductance and Ca^2+^ permeability. The dichotomy is reinforced by the observation that the anomalous current is absent in cochlear cells of *Piezo2*‐null mice, even though the normal MT current persists. The anomalous current is suppressed by high intracellular Ca^2+^, suggesting that influx of the divalent ion via more Ca^2+^‐permeable normal MT channels inhibits the anomalous channels, thus explaining the temporal relationship between the two. *Piezo2*‐null mice have largely normal hearing, exhibiting up to 20 dB elevation in threshold in the acoustic brainstem response, so raising questions about the significance of PIEZO2 in the cochlea. Since the anomalous current declines with postnatal age, PIEZO2 may contribute to hair cell development, but it does not underlie the normal MT current. Its role in the development of hearing is not understood.

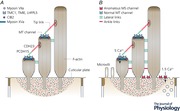

## Introduction

One of the most significant recent advance in physiology has been the identification of PIEZO as a pore‐forming subunit of vertebrate mechanically gated ion channels (Coste *et al*. 2012). The discovery was rendered more remarkable by the demonstration that one or other of the two isoforms PIEZO1 or PIEZO2 underlies transduction in most cutaneous mechanoreceptors and muscle spindles (Ranade *et al*. 2014; Woo *et al*. 2014, 2015) as well as in other tissues such as blood vessels and bone. The discovery of PIEZO marks a watershed event after a protracted search for the ion channel underpinning vertebrate mechanosensation. Until recently, knowledge about this channel type has been sparse compared to information available for the two other canonical channel types, voltage‐gated and ligand‐gated channels. The discovery has revealed a large set of human genetic disorders originating from mutations in *PIEZO1* or *PIEZO2* (Alper, 2017). One organ in which mechanosensation is central to physiological function is the inner ear, where sensory hair cells detect sound stimuli in the cochlea and head motion in the labyrinth. It is natural to enquire whether hair cell transduction also relies on PIEZO channels. The quest for the hair cell mechanotransducer channel has been long, and hindered by the paucity of tissue available for biochemical analysis (Fettiplace & Kim, 2014).

Hair cell transduction occurs in the bundle of so‐called hairs or stereocilia protruding from the top of the cell in an organ pipe arrangement. Adjacent actin‐filled stereocilia are connected by extracellular filaments, the most important of which is the tip link, extending from the top of one stereocilium to the side wall of its taller neighbour (Pickles *et al*. 1984; Furness *et al*. 2008). Sub‐micrometre deflections of the hair bundle tension the tip links and gate mechanotransducer (MT) channels at their lower end (Howard & Hudspeth, 1988; Beurg *et al*. 2009). Normal transduction is functionally polarized, reflecting the morphological asymmetry of the bundle (Shotwell *et al*. 1981; Beurg *et al*. 2016). In outer hair cells (OHCs) of the mammalian cochlea, an indicator of bundle polarity is the ‘V’ shape. Deflection of the hair bundle towards its taller edge to the tip of the ‘V’ increases tension in the tip links and opens MT channels, whereas deflection towards its shorter edge relaxes the tip links, hence closing the MT channels. Some of the protein constituents of the transduction apparatus are known. The tip link is composed of dimers of cadherin 23 and of protocadherin 15 joined at their N‐termini (Kazmierczak *et al*. 2007). Some other components have also been identified from genetic mutations (Abstract figure panel *A*) (Richardson *et al*. 2011; Fettiplace & Kim, 2014), but controversy has persisted over the identity of the transducer channel and, as discussed later, it is unlikely to be an isoform of PIEZO. The current candidate is a transmembrane channel‐like protein isoform 1 and/or 2 (TMC1, TMC2) (Kawashima *et al*. 2011; Kim & Fettiplace, 2013; Pan *et al*. 2013). The two C‐termini of protocadherin 15 lie at the bottom end of the tip link where they are thought to interact with multiple membrane proteins: TMC1/2 (Maeda *et al*. 2014), LHFPL5 (Xiong *et al*. 2012), TMIE (Zhao *et al*. 2014) and CIB2 (Giese *et al*. 2017). These proteins, probably with others still unknown, are thought to form the mechanotransduction channel complex.

## Anomalous currents

Key evidence for a role for TMC1 in hair cell transduction is the many mutations of the *Tmc1* gene linked to deafness in humans and in mice. *Tmc1* knockout in mice also results in loss of the hair cell MT current, though in the early postnatal period the transient expression of *Tmc2* can substitute for *Tmc1*. Surprisingly, in *Tmc1:Tmc2* double knockouts, the normal MT current is totally absent from cochlear hair cells but, paradoxically, a ‘reverse‐polarity’ mechanosensitive (MS) current can still be recorded in such mutants (Kim *et al*. 2013). It was initially considered that the ‘reverse‐polarity’ current might be a manifestation of the normal current but this turned out not to be the case. The ‘reverse‐polarity’ current was so named because it could apparently be evoked by hair bundle deflections in the non‐optimal direction, towards the shortest rank of stereocilia. However, use of this term is misleading, because it is now thought to be activated by deformation of the apical plasma membrane (Abstract figure panel *B*) rather than a specific direction of deflection of the hair bundle (Beurg *et al*. 2016), and it will subsequently be referred to as an anomalous MS current.

**Figure 1:**
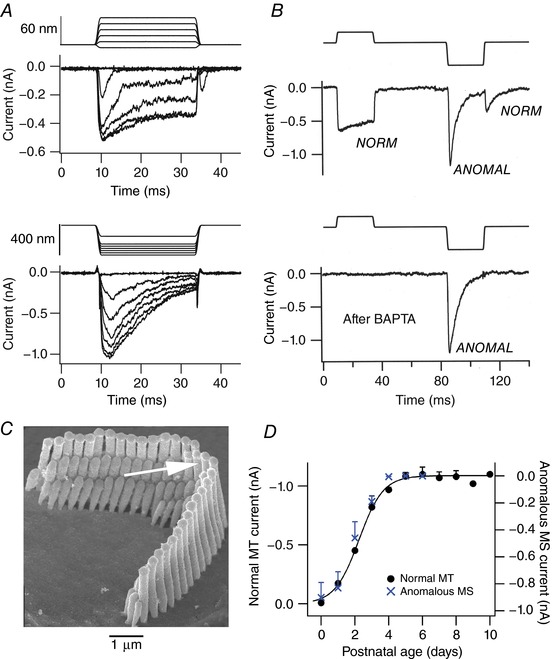
Normal and anomalous (reverse‐polarity) currents in mouse cochlear hair cells *A*, in a wild‐type OHC, both a normal MT current (above) and anomalous MS current (below) can be evoked by mechanical stimuli. Shown above each current family is the bundle motion, the saturating range being smaller for normal MT currents than for anomalous MS currents. *B*, both normal (*NORM*) and anomalous (*ANOMAL*) currents can be recorded in the same wild‐type hair cell at postnatal day 1. Treatment with BAPTA, which severs the tip links, causes loss of the normal but not the anomalous (*ANOMAL*) current. *C*, scanning electron micrograph of mouse OHC stereociliary bundle. Arrow denotes direction of optimal stimulus towards taller edge of bundle, which opens MT channels. Anomalous currents evoked by displacements in opposite direction to arrow. *D*, growth of normal MT current (filled circles) is synchronized with decline of anomalous MS current (crosses) with early development in wild‐type apical OHCs. Note anomalous current axis is reversed and scaled. Results are expressed as mean ± SEM for five or more cells and are modified from Kim *et al*. (2013) and Beurg *et al*. (2016).

Such anomalous currents can be recorded in cells in which normal transduction has, for various reasons, been lost. The most common cause is the loss of the tip links, resulting from various manipulations or conditions including: (i) mutations in the tip link constituent protocadherin‐15 (Alagramam *et al*. 2011); (ii) lowering Ca^2+^ in the saline bathing the hair bundle to less than 1 μm, usually by applying BAPTA (Kim *et al*. 2013; Marcotti *et al*. 2014), which destroys the tip links (Assad *et al*. 1991); (iii) some mutations of the myosin VIIa, which is part of the tip link's upper attachment point (Kros *et al*. 2002; Marcotti *et al*. 2014); (iv) absence of the calcium and integrin binding protein CIB2, thought to be complexed with TMC1 at the tip of the stereocilia (Giese *et al*. 2017); and (v) mutations of other proteins linked with mechanotransduction, such as TMIE (Zhao *et al*. 2014). In each case, the normal MT current is absent and is replaced by the anomalous MS current. In two other mutants, anomalous currents have been recorded, along with normal MT currents. These are in the *Shaker2* mutation in myosin XVa (Stepanyan & Frolenkov, 2009), and in the knockout of *Vlgr1* in which the protein product is a component of the ankle links (Michalski *et al*. 2007). The anomalous currents in these two mutants have not been examined in detail, and it is unclear whether they are the same phenomenon as seen in other mutants where normal transduction is lost. However, there is one situation during neonatal development in wild‐type animals in which both the normal MT current and anomalous MS current are present at the same time (Fig. 1) (Waguespack *et al*. 2007; Kim *et al*. 2013; Marcotti *et al*. 2014; Beurg *et al*. 2016). Here, the anomalous MS current appears first around birth, but is down‐regulated as the normal MT current grows. Strikingly, the changes in the two currents are synchronized so that reduction in the anomalous current is contemporaneous with growth in the normal current (Fig. 1). This relationship between the two types of mechanosensitive current occurs in all inner ear hair cells of wild‐type animals, inner hair cells (IHCs) and OHCs of the cochlea, and vestibular hair cells (Beurg *et al*. 2016). Furthermore, the timing of these changes depends on the cell types. For example, the changes in both anomalous and normal currents in basal OHCs precede those changes in apical OHCs by about 2 days, reflecting a base to apex gradient in hair cell differentiation; in vestibular hair cells, changes in anomalous and normal currents both occur prenatally (Beurg *et al*. 2016). Whether present neonatally in wild‐type animals, or initiated by loss of normal transduction, the anomalous MS current has usually been found to decline over development with a similar time course. However, it should be noted that not everyone has reported a strict temporal relationship between the normal and anomalous currents (Marcotti *et al*. 2014). A possible synchronization between the two currents raises the prospect that they are causally related.

## Single stretch‐sensitive channels

**Figure 2:**
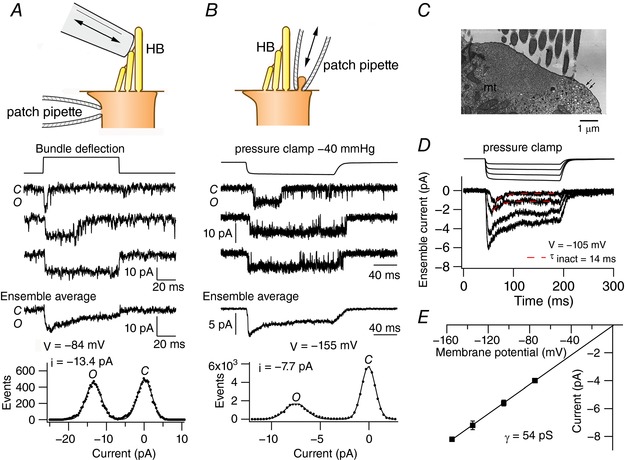
Mechanosensitive channels in OHCs *A*, normal MT channels recorded under whole‐cell conditions after destroying almost all tip links with BAPTA, recording and stimulation methods shown at the top. HB, hair bundle. Three records of single‐channel activity for a positive hair bundle deflection, ensemble average displaying adapting and sustained components of activity, and amplitude histogram indicating a 13.5 pA channel; at the membrane potential of −84 mV, this current corresponds to unitary conductance of 160 pS. Mid‐region of cochlea, hair bundle bathed in Na^+^, 0.04 mm Ca^2+^. *B*, stretch‐activated channel recorded in cell‐attached patch on hair cell apical membrane, method depicted above. Three records of single‐channel activity for a negative pressure step imposed on membrane patch via pipette; ensemble average shows transient and sustained activity, and amplitude histogram of traces, indicating a 7.7 pA channel at −155 mV, equivalent to 51 pS conductance. Pipette solution Na^+^, 0.04 mm Ca^2+^. *C*, section of apical region of hair cell where cell‐attached recordings made; note secretory vesicles (arrowheads), exocytotic pits (arrows) and microtubules (mt). *D*, ensemble‐average currents of stretch‐activated channel in *B* at four pressure levels at −105 mV. Time constant of inactivation, τ_inact_, is 14 ms for the smaller stimuli. *E*, dependence of current for stretch‐activated channel on holding potential, linear fit gives conductance 54 pS. Results modified from Beurg *et al*. (2016).

The prevailing view is that the anomalous current is evoked not by bundle displacement *per se* but rather deformation of the apical cell membrane (Beurg *et al*. 2016). Anomalous currents evoked with different orientations of a fluid jet indicate that the best stimulus is suction on the membrane from whatever angle, rather than polarized deflection of the hair bundle. Thus if a sinusoidal stimulus was applied, the current was activated on the negative (suction) phase of the fluid jet. Evidence derived from Ca^2+^ imaging and from single‐channel recording then argued that the underlying MS channels are located on the apical cell membrane at the base of the hair bundle. They might also exist in the stereociliary membrane but the experimental assays are insufficiently sensitive to draw this conclusion. Cell‐attached patches on the apical membrane, lateral to the hair bundle, showed clear synchronization of channel activity to membrane suction applied via the patch pipette with a fast pressure clamp. An example of their activity, compared with that of a normal MT channel, is shown in Fig. 2. After the onset of the stimulus, there was an initial increase in channel activity that subsequently declined, indicative of inactivation. In recordings from OHCs of neonatal wild‐type mice, the mean channel conductance was 53 ± 3 pS (*n* = 8). Current–voltage relationships were linear with an extrapolated reversal potential close to 0 mV, consistent with a cation channel. Single stretch‐activated channels were also present in *Tmc1:Tmc2* double knockouts and had a mean conductance of 52 ± 1 pS (*n* = 5), not significantly different from the value in wild‐type animals. The ensemble averages showed an inactivating component with a time constant dependent on stimulus level, increasing from a minimum of ∼14 ms for the smallest stimuli up to 30 ms for maximal saturating stimuli (Fig. 2 *D*). The adaptation time constant was weakly voltage sensitive, but it was unaffected by external Ca^2+^ concentration (Kim *et al*. 2013). The recordings in Fig. 2 *B* were made in a low external Ca^2+^ (0.04 mm), comparable to the concentration of Ca^2+^ in the endolymph bathing the hair bundles *in vivo* (Gill & Salt, 1997). When an external Ca^2+^ concentration of 1.5 mm, similar to that in perilymph, was used in the patch pipette, the channel conductance was reduced to 34 ± 1 pS (*n* = 5), demonstrating block by external Ca^2+^ similar to the block seen with normal MT channels (Fettiplace & Kim, 2014).

The anomalous MS channels were initially considered to be a possible candidate for the normal MT channel. While the properties of the anomalous channel are similar to the normal MT channel, they are not identical. The latter have been characterized principally from whole‐cell recordings after destruction of the majority of tip links by exposure to sub‐micromolar Ca^2+^ and buffered with BAPTA, which is thought to leave a few functional MT channels. This approach is successful because the underlying channels have large conductance, 100 pS or more (Geleoc *et al*. 1997; Ricci *et al*. 2003; Beurg *et al*. 2006), and can be seen above the noise imposed by the series resistance of the patch pipette (Fig. 2 *A*). Differences between the anomalous MS channels and normal MT channels include distinct single‐channel conductance, Ca^2+^ permeability and differential susceptibility to a range of pharmacological blocking agents (Table 1). In particular, the IC_50_ for dihydrostreptomycin is one to two orders of magnitude larger for the anomalous MS currents than for the normal MT currents (Marcotti *et al*. 2014; Beurg *et al*. 2014). These differences have prompted them to be assessed as distinct channels (Marcotti *et al*. 2014). It should be noted, however, that the IC_50_ for another blocking agent, amiloride, at ∼50 μm, is similar between the two channel types (Rusch *et al*. 1994; Beurg *et al*. 2014). Furthermore, GsMTx4, a peptide toxin of the tarantula spider, *Grammostola spatulata* (Suchyna *et al*. 2000), blocks both normal and anomalous MS channels with similar sub‐micromolar IC_50_ (Beurg *et al*. 2014). This peptide also blocks the PIEZO1 mechanosensitive channel (Bae *et al*. 2011).

**Table 1:** Comparison of properties of normal MT channels/currents and anomalous MS channels/currents

Property	Normal MT channels	Anomalous MS channels
Ca^2+^ permeability *P*_Ca_:*P*_Cs_	5:1	1.5:1
Single‐channel conductance (Na^+^, low Ca^2+^)	>100 pS	50–60 pS
Dihydrostreptomycin IC_50_ a	11 μm	169 μm,
Dihydrostreptomycin IC_50_ b	7 μm	894 μm
FM1‐43 IC_50_	2.8 μm	26 μm
Amiloride IC_50_	50 μm	58 μm
*Piezo2* knockout	Unaffected	Absent

IC_50_ for blocking agents all given for holding potential of −84 mV. Results from Kim & Fettiplace (2013); Beurg *et al*. (2014, 2016); Wu *et al*. (2017). The two values for dihydrostreptomycin were: ^a^Beurg *et al*. (2014); ^b^Marcotti *et al*. (2005, 2014). Control values for amiloride from Rusch *et al*. (1994).

## The anomalous channels are PIEZO2

**Figure 3:**
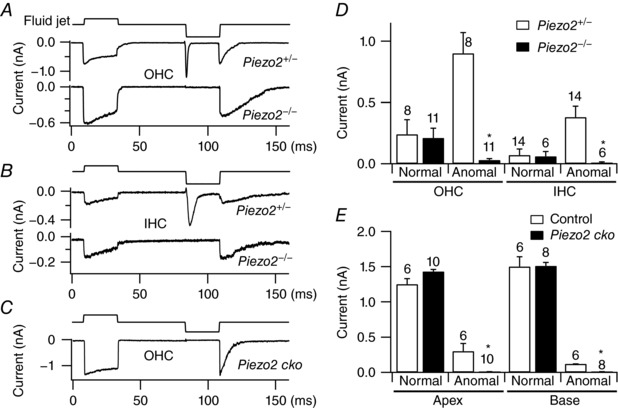
Effects of *Piezo2* knockout on normal and anomalous currents in cochlear hair cells *A*, normal MT and anomalous (reverse‐polarity) MS currents in OHCs of *Piezo2* heterozygote (top) and *Piezo2* homozygote (bottom) in P0–P1 mice. *B*, normal MT and anomalous MS currents in IHCs of *Piezo2* heterozygote (top) and *Piezo2* homozygote (bottom) in P0–P1 mice. *C*, normal polarity MT but no anomalous MS currents seen in *Piezo2* P2 conditional knockout (*Piezo2 cko*). *D*, collected results of normal and anomalous currents in *Piezo2* heterozygotes and homozygotes, mean ± SEM and number of cells. For anomalous currents in both OHCs (left) and IHCs (right), means were significantly reduced (^*^ *P* < 0.001, Student's *t* test) in homozygote relative to the heterozygote, but the mutation had no effect on normal‐polarity current. *E*, collected results of normal and anomalous currents on *Piezo2* P2–P4 conditional knockouts in apical and basal OHCs.

The functional relationship between the normal and anomalous mechanosensitive currents is unclear, but recent work has verified that they have distinct protein identities (Wu *et al*. 2017). In particular, the anomalous MS current is lost in knockouts of *Piezo2* but the normal MT current is unaffected by such mutants. Thus PIEZO2 is required for the anomalous currents observed during hair cell development in both OHCs and IHCs, but it is not an essential component of the normal MT channel in either cell type (Fig. 3). Furthermore, PIEZO2 labelling has been shown to occur on the apical membrane of cochlear and vestibular hair cells, near the junction with their supporting cells (Wu *et al*. 2017), which is consistent with the location of the stretch‐sensitive channels recorded in cell‐attached patches. Comparison of the properties of PIEZO2 channels and channels carrying anomalous MS currents in hair cells demonstrates a close agreement. Thus, both are cation channels with comparable Ca^2+^ selectivity: *P*_Ca_/*P*_Cs_, ∼1.2 in PIEZO1 (Coste *et al*. 2015) and ∼1.8 for anomalous channels (Beurg *et al*. 2014). Both are blocked by FM1‐43 with IC_50_ of ∼9 μm (PIEZO1; Eijkelkamp *et al*. 2013) and 26 μm (anomalous channels: Beurg *et al*. 2016) and exhibit fast inactivation (Kim *et al*. 2013; Coste *et al*. 2015). Unitary conductances for the PIEZO2 channel and for the anomalous MS channel are also similar: for PIEZO2, the values are 24 pS (1 mm Ca^2+^, 1 mm Mg^2+^) and 57 pS (Ca^2+^ and Mg^2+^ free) (Coste *et al*. 2015); for the anomalous MS channel, they are 34 pS (1.5 mm Ca^2+^, 0 mm Mg^2+^) and 53 pS (0.04 mm Ca^2+^, 0 mm Mg^2+^), respectively. For both channel types, the difference in conductance in the high and low extracellular Ca^2+^ indicates a similar 40–50% block by external divalents. PIEZO2 channels expressed in HEK293 cells showed no inhibition by 10 mm intracellular Ca^2+^ (Wu *et al*. 2017), suggesting that fast block of these channels by divalents (Coste *et al*. 2012) occurs at an external site. The functional similarity of the anomalous MS channels characterized in hair cells, and the PIEZO2 channels assayed in heterologous cell types, reinforces the conclusion from the *Piezo2* knockouts.

## Role of PIEZO2 in hair cells

Over the past 15 years, substantial information about the molecules underlying development and function of the inner ear hair cells has accrued from study of mutations in both humans and mice (Richardson *et al*. 2011). What role does early expression of *Piezo2* play in hearing? *Piezo2* knockout throughout the inner ear led to an approximately 15 dB threshold elevation of auditory brainstem response, and to a mild distortion of auditory signal, with no vestibular defect (Wu *et al*. 2017). OHC electromotility, as assayed by non‐linear capacitance, was unaltered, suggesting no defect in prestin‐mediated cochlear amplification. Since the *Piezo2* null is embryonic lethal due to failure of respiration, these measurements were performed on conditional knockout mice, by crossing floxed *Piezo2* with *Pax2 Cre*. But the role of PIEZO2 in hearing is obscure.

What is clear is that the anomalous currents, presumably reflecting PIEZO2 channels, are present during early development in all mammalian hair cell types, both auditory and vestibular. Moreover, their down‐regulation is synchronized with the appearance of normal mechanotransduction (Fig. 1 *D*). It is important to note that both types of mechanosensitive channel are permeable to Ca^2+^, but the normal MT channel is threefold more permeable, and also partially open at rest, so permitting a sustained Ca^2+^ influx (Beurg *et al*. 2014). The anomalous MS current has been shown to be inhibited by raising intracellular Ca^2+^ (Wu *et al*. 2017), but a concern with this experiment is that an unrealistically high Ca^2+^ concentration (1 mm) was needed to accomplish the block. In some cells, significant block could be achieved with 0.35 mm Ca^2+^. This high concentration may not be unrealistic, since Ca^2+^ in the stereocilia has been estimated from reversal potential measurements to be near‐millimolar levels (Beurg *et al*. 2015). Although Ca^2+^ will be highest near the inner face of the MT channel, the concentration further down the stereocilium, and at the top of the cell, may also be substantial, as Ca^2+^ buffering and extrusion in neonates is small (Hackney *et al*. 2005; Chen *et al*. 2012). Furthermore, the Ca^2+^ concentration in endolymph bathing the hair bundle is likely to be high, millimolar, levels prior to the emergence of the endolymphatic potential after the first postnatal week. Thus, during neonatal development, Ca^2+^ influx through the more Ca^2+^‐permeable normal MT channels may elevate Ca^2+^ under the apical membrane to a level sufficient to suppress the anomalous current. This could explain the apparent developmental relationship between the two. The Ca^2+^‐induced inhibition is unlikely to be due to direct intracellular blockade as it takes time to develop, so it may involve some other signalling cascade. Furthermore, as noted above, PIEZO channels expressed in HEK293 cells showed no inhibition by 10 mm intracellular Ca^2+^ (Wu *et al*. 2017). These observations argue that the PIEZO2 channels are indeed downregulated by high intracellular Ca^2+^, rather than just being blocked. Since the anomalous current also appears during damage to hair cell transduction, it is possible that PIEZO2 contributes to repair of damaged hair cells or their apoptosis during ageing. But the presence of these channels in early neonates suggests they may also play a developmental role.

## Concluding remarks

It is puzzling that the PIEZO1 or PIEZO2 channels, which are ubiquitous in mediating transduction of mechanical forces in the majority of vertebrate mechanoreceptors, are not employed as the primary MT channel in hair cells. This is particularly perplexing when PIEZO2 is known to occur in hair cells, and to have properties comparable, in conductance, ionic selectivity and adaptation, to those of the native MT channel. A difference in Ca^2+^ selectivity may be crucial: *P*_Ca_/*P*_Cs_ = 1.8 for the anomalous PIEZO2 channels (Kim *et al*. 2013) but *P*_Ca_/*P*_Cs_ = 5 for the MT channel in OHCs (Kim & Fettiplace, 2013). Ca^2+^ influx through the hair cell channel is essential for generating adaptation, and has also been proposed to be necessary for regulating actin polymerization at the stereociliary tips and hair bundle organization (Velez‐Ortega *et al*. 2017). This requirement is presumably the reason that, in order to direct bundle maturation, cochlear hair cells acquire mechanotransducer channels early in development, well before the onset of hearing, which occurs around postnatal day 12 in mice. The kinetics of activation and adaptation are fairly rapid for PIEZO channels and for the anomalous MS currents, but whether these are sufficiently fast to meet the sub‐millisecond time constants needed to encode acoustic stimuli up to 70 kHz in mice is unclear. A property that may be relevant is the mode of channel activation. PIEZO1 channels reconstituted in lipid bilayers can be activated by tensional forces in the bilayer in the absence of any other cellular components (Coste *et al*. 2012; Syeda *et al*. 2016). Direct force sensation, mediated via the lipid bilayer, has also been demonstrated for the bacterial MscL channel (Teng *et al*. 2015; Rosholm *et al*. 2017). However, despite modelling (Kim, 2015), there is no experimental evidence that the hair cell MT channel can be similarly activated. An alternative mechanism is for the channel to be tethered to the cytoskeleton, exemplified by NOMPC, which is thought to be anchored to cytoplasmic microtubules via an extended N‐terminus comprising multiple ankyrin repeats (Zhang *et al*. 2015). To be ultra‐sensitive with rapid activation may necessitate the MT channel to be gated by forces delivered across a protein core that is tethered on both the extracellular and intracellular faces of the membrane. Such anchors may involve special proteins such as protocadherin 15 in the tip link, and TMIE, LHFPL5 and CIB2 in the cytoplasm. Together these attachments may underlie the ‘gating spring’ that has been postulated from hair bundle mechanical measurements (Howard & Hudspeth, 1988). It is therefore conceivable that the hair cell MT channel cannot be gated by tension in the bilayer. An important implication is that activation of any candidate protein by stretching the lipid membrane of a heterologous expression system may be unsuccessful. This could be the reason that evidence for TMC1 as the MT channel pore is lacking. But at this stage, the molecular identity of the normal MT channel remains controversial, as is the functional significance of the anomalous MS channel.

## Additional information

### Competing interests

None declared.

### Author contributions

Both authors contributed to experiments, analysis of results and writing of the manuscript.

### Funding

The work was funded by grants RO1 DC01362 and DC015439 to R.F. from the National Institutes on Deafness and other Communication Disorders.
